# Axiom Microbiome Array, the next generation microarray for high-throughput pathogen and microbiome analysis

**DOI:** 10.1371/journal.pone.0212045

**Published:** 2019-02-08

**Authors:** James B. Thissen, Nicholas A. Be, Kevin McLoughlin, Shea Gardner, Paul G. Rack, Michael H. Shapero, Raymond R. R. Rowland, Tom Slezak, Crystal J. Jaing

**Affiliations:** 1 Physical & Life Sciences Directorate, Lawrence Livermore National Laboratory, Livermore, California, United States of America; 2 Computation Directorate, Lawrence Livermore National Laboratory, Livermore, California, United States of America; 3 Thermo Fisher Scientific, Santa Clara, California, United States of America; 4 Department of Diagnostic Medicine and Pathobiology, Kansas State University, Manhattan, Kansas, United States of America; Hokkaido University, JAPAN

## Abstract

Microarrays have proven to be useful in rapid detection of many viruses and bacteria. Pathogen detection microarrays have been used to diagnose viral and bacterial infections in clinical samples and to evaluate the safety of biological drug materials. In this study, the Axiom Microbiome Array was evaluated to determine its sensitivity, specificity and utility in microbiome analysis of veterinary clinical samples. The array contains probes designed to detect more than 12,000 species of viruses, bacteria, fungi, protozoa and archaea, yielding the most comprehensive microbial detection platform built to date. The array was able to detect *Shigella* and *Aspergillus* at 100 genome copies, and vaccinia virus DNA at 1,000 genome copies. The Axiom Microbiome Array made correct species-level calls in mock microbial community samples. When tested against serum, tissue, and fecal samples from pigs experimentally co-infected with porcine reproductive and respiratory syndrome virus and porcine circovirus type 2, the microarray correctly detected these two viruses and other common viral and bacterial microbiome species. This cost-effective and high-throughput microarray is an efficient tool to rapidly analyze large numbers of clinical and environmental samples for the presence of multiple viral and bacterial pathogens.

## Introduction

Rapid detection and characterization of bacterial and viral pathogens is important for clinical microbiological diagnostics, public health, veterinary diagnostics, drug and food safety, environmental monitoring and biodefense. Various detection technologies based on nucleic acid signatures have emerged in the past few years, including TaqMan PCR [1] and Luminex bead-based systems [2, 3]. While these technologies can rapidly identify selected pathogens at the species or strain level, they do not have the capability to provide a holistic view of microorganisms present in analyzed samples. Characterization of known and emerging pathogens requires a platform that has the capacity to assess whole genome sequence content from a variety of pathogens very rapidly. While metagenomic sequencing provides the most in-depth information to characterize a microbial genome, the costs, labor, and time associated with library preparation, sequencing, bioinformatic analysis, and data storage may be prohibitive when analyzing many samples in a standard laboratory setting.

The Lawrence Livermore Microbial Detection Array (LLMDA) is a comprehensive microbial detection chip that includes probes to identify over a wide range of microbes including viruses, bacteria, fungi, protozoa and archaea. The initial version of the LLMDA (v.2) covered ~2,200 bacterial and ~900 viral species with complete genome sequences available as of spring 2007 [4–10]. The NimbleGen 1-plex 388K array was the platform of LLMDA v.2. The array was used to determine contaminants in vaccines, clinical diagnostics, identifying pathogens in cancer [4–10]. A 4-plex 72K version of the array was also designed using a subset of the probes from LLMDA v.2. The sensitivity of the array was evaluated using bacterial and viral pathogens [11]. Subsequently, the LLMDA v.5 was designed to target 3,521 vertebrate-infecting species including1,856 bacteria, 1,398 viruses, 48 fungi, 94 protozoa and 125 archaea species (current as of December 2011). The LLMDA v.5 used the Roche NimbleGen 12-plex array with 135K probes per subarray. The v.5 array was applied in the detection of bacteria from plague victims, profiling pathogen from combat wound samples, detection of pathogens from lung cancer and lymphomas, and in pathogen surveillance of wild dolphins [12–17]. The LLMDA v.7 was expanded to detect a total of 8,101 species of microbes including 3,856 viruses, 3,855 bacteria, 254 archaea, 100 fungi and 36 protozoa (current as of June 2013). The LLMDA v.7 used the Agilent 4-plex 180K format of the array and the array was applied to veterinary diagnostics [18, 19].

One main limitation for the wider use of pathogen detection microarrays is the cost of the assay, which is at least an order of magnitude higher than PCR assays [20]. To fill this technology gap, the Axiom Microbiome Array, a cheaper and higher-throughput microbial detection array platform was recently designed. The Axiom array platform has been widely used for genotyping applications to detect human, plant and animal single nucleotide polymorphisms [21–23]. The Axiom Microbiome Array is the first microarray design for microbiome analysis using the high-density Axiom platform. The Axiom Microbiome Array contains a total of 1.38 million DNA probes to analyze the specific species in a microbiome or other complex sample. The array processes 24 or 96 samples in parallel on a 96-well plate format. The cost of one sample processed on the array is now closer to the cost of a PCR or multiplex PCR test [24]. Axiom Microbiome Array is cheaper than metagenomic DNA sequencing and provides an alternate method for profiling microbiological contents in complex clinical and environmental samples. A comparison of metagenomic sequencing, 16S rRNA sequencing and Axiom Microbiome Array in resolution, speed and cost is shown in Table 1.

**Table 1:** Comparison of Axiom Microbiome Array vs 16S rRNA sequencing vs metagenomic sequencing.

Features	16S rRNA sequencing	Metagenomic sequencing	Axiom Microbiome
**Detect sequenced bacteria**	Y	Y	Y
**Detect sequenced viruses**	N	Y	Y
**Detect sequenced fungi, archaea, protozoa**	N	Y	Y
**Detect un-sequenced microorganisms**	Y (bacteria only)	Y	N
**Detect functional genes**	N	Y	N
**Species resolution**	N	Y	Y
**Strain resolution**	N	Y	N
**What is detected**	16S rRNA gene	Genome fragments	Genome fragments
**Length of DNA detected (DNA bases)**	~400	~150–300	~35
**Instruments**	Illumina MiSeq, Thermo Fisher Ion Torrent	Illumina NextSeq, NovaSeq, HiSeq	Thermo Fisher GeneTitan
**Instrument cost**	++	++++	+++
**Time on instrument (hours)**	24–36	26–264	48
**Per-sample reagent cost**	$30–50	$300–500	$40–50

A series of experiments with the Axiom Microbiome Array was performed to estimate the minimum detectable concentrations of single viral, bacterial and fungal templates and mixtures of bacterial agents in a microbial community. In addition, the array was tested using several types of animal clinical samples (serum, tonsil, fecal) to evaluate its utility in veterinary diagnostics and pathogen surveillance.

## Methods

### Probe design for the Axiom Microbiome Array

Axiom Microbiome Array harbors a total of 1.38 million probes on each array. These include 1,277,846 target probes and 60,152 random negative control probes. The target probes represent 135,555 sequences (genomes, contigs, segments, or plasmids) from 12,513 microbial species current as of October 2014, as indicated in Table 2. The probe sequences are available from Thermo Fisher as part of the MiDAS (Axiom Microbial Detection Analysis Software) analysis package. The Axiom arrays utilize 35-mer oligonucleotide probes synthesized in situ on a microarray substrate, with automated, parallel processing of 96 samples per plate. Features are 3 μm squares, at a pitch of 5 μm center-to-center. The arrays were ordered from Thermo Fisher Scientific as a commercially available product.

**Table 2:** Probe summary for Axiom Microbiome Array. The number of microbial sequences were current as of October 2014.

Domain	Number of families	Number of species	Number of target sequences
**Archaea**	31	370	606
**Bacteria**	278	6901	34,254
**Fungi**	121	381	658
**Protozoa**	30	91	229
**Viruses**	100	4770	99,808
**Total**	560	12,513	135,555

### DNA sample acquisition and quantitation

Vaccinia Lister DNA was obtained from Advanced Biotechnologies Inc. (Columbia, MD) and copy number quantitation was performed by Advanced Biotechnologies using real-time PCR on the Roche LightCycler. *Shigella flexneri* strain 24570 (ATCC 29903) and *Aspergillus fumigatus* strain 118 (ATCC 1022) DNA were obtained from internal sources at Lawrence Livermore National Laboratory (LLNL). DNA concentration was determined by Qubit spectrophotometer and the DNA genome copy numbers per test sample were calculated based on the genomic size of the two bacteria. *S*. *flexneri*, *A*. *fumigatus* and Vaccinia Lister DNA were 10-fold serially diluted from 10,000 copies to 10 copies. Soil was collected from locations in San Francisco as described previously [25] and DNA was extracted using the MoBio PowerSoil extraction kit (Catalog number: 12888–50). Soil DNA was quantified using the Qubit spectrophotometer and each quantity of microbial DNA was spiked into 1.0 ng of soil DNA prior to whole genome amplification in the Axiom microarray process.

The Metagenomic Control Material for Pathogen Detection was obtained from ATCC (ATCC MSA-4000). This is a mock microbial community used as a microbiome standard that mimics metagenomic samples. The product mix comprises of 11 bacterial DNAs from fully sequenced genomes, making this a good sample to test on the Axiom Microbiome Array. The percentage of each DNA within the mix varies from 0.1% to 28.9% by mass. The concentration of each genomic DNA in the mixture was between 0.80 ng/μL and 1.33 ng/μL.

A second genomic mix from ATCC, the 20 Strain Staggered Mix Genomic Material (ATCC MSA-1003) was also used to test the array detection dynamic range. Just like the ATCC MSA-4000, this is a mock microbial community often used as a microbiome standard that mimics metagenomic samples. This genomic DNA mix contains 20 genomic DNAs from fully sequenced genomes that were selected based on relevant phenotypic and genotypic attributes, such as Gram stain, GC content, genome size, and spore formation. The concentration of the DNA in this panel span four orders of magnitude ranging from 0.02% to 18% by mass in the final mixture. The concentration of each genomic DNA in the mixture was between 6.08 ng/μL and 10.14 ng/μL.

### Pig serum, tonsil and fecal sample extraction

All use and experimentation incorporating animals and viruses were done in accordance with the Federation of Animal Science Societies (FASS) Guide for the Care and Use of Agricultural Animals in Research and Teaching, the USDA Animal Welfare Act and Animal Welfare Regulations, and approved by the Kansas State University Institutional Animal Care and Use Committees and Institutional Biosafety Committees. Pig serum and tonsil samples were obtained from a project to evaluate the role of host genetics using pigs experimentally co-infected with porcine reproductive and respiratory syndrome virus (PRRSV) and porcine circovirus type 2 (PCV-2). The detailed animal care was described in this previous study [18]. DNA and RNA from serum and tonsil tissues were extracted using Trizol LS reagent as described previously [18]. DNA from fecal samples were extracted using the PowerViral Environmental RNA/DNA Isolation Kit (Qiagen) as described [17]. All DNA samples were quantitated using Qubit fluorometer. For serum samples, 0.1, 0.2 and 2 ng was used and for tonsil samples, 32, 34 and 41 ng were used for array experiments. The fecal samples were run in triplicates on the microarray.

### Axiom chemistry and microarray hybridization

The Axiom Microbiome Array was run using the Axiom 2.0 Assay biochemistry which includes an isothermal whole-genome amplification step per the manufacturer’s directions. For RNA samples, a reverse transcription reaction was performed with Superscript VILO per the guidance in the Axiom Microbiome Array User Guide. cDNA derived from these samples were used as a substrate for the Axiom whole genome amplification. No DNase treatment was employed on the RNA samples, therefore any DNA from the total nucleic acid preparations will be carried over in the whole genome amplification. The array was hybridized, washed and scanned on the GeneTitan Multi-Channel instrument in an automated fashion according to manufacturer’s instructions (Thermo Fisher, Waltham, MA). Both the 96-well and 24-well plates were used to run the various clinical and spiked samples to evaluate the array’s sensitivity and applicability in clinical samples.

### Microarray data analysis

Microarray data were analyzed using the MiDAS software (Axiom Microbial Detection Analysis Software) (Thermo Fisher) which is based on the Composite Likelihood Maximization Method (CLiMax) algorithm developed at LLNL [4, 26]. Axiom MiDAS performs single-sample analysis of CEL files from Axiom Microbiome Arrays and automatically generates a comprehensive analysis summary in a simple-to-use software package. Probes with signal intensity above the 99^th^ percentile of the random control probe intensities and with more than 20% of target-specific probes detected were considered positive. MiDAS uses initial and conditional scores to determine the likelihood of the target presence. The initial score is the log likelihood ratio for the target being present in the sample if no other targets are present, vs no targets being present in the sample. This value gives information on what the maximum possible contribution of that target is to the holistic model of the sample, based on the probes observed when interrogating a sample with the Axiom Microbiome Array. The conditional score gives an indicator of the actual contribution of each target to the model of the sample; it is the log likelihood for a model including the target vs for a model without the target. As the conditional score takes into account the presence of other targets, it can be lower than the initial score for a given target if there are probes in common between targets.

### Reproducibility analysis

Reproducibility on Axiom Microbiome Array was assessed using two metrics: the consensus hit rate and the consensus precision rate. These methods were developed for the assessment of the reproducibility of the microbial detection calls from unknown samples. Target sequences are considered present in the sample if the conditional score is > 0 and the ratio of conditional to initial score is > 0.2. For the metrics below, the numerator (# of sample calls matching consensus calls) is defined as the number of calls by MiDAS that meet the criterion of being identified in over 50% of the replicate samples tested.

$$Consensus\;Hit\;Rate=\frac{\#\;of\;sample\;calls\;matching\;consensus\;calls}{total\;\#\;of\;consensus\;calls\;(across\;all\;samples)}$$

$$Consensus\;Precision\;=\;\frac{\#\;of\;sample\;calls\;matching\;consensus\;calls}{total\;\#\;of\;sample\;calls}$$

## Results

### Detection sensitivity of *Shigella flexneri*, *Aspergillus fumigatus* and vaccinia virus

Table 3 shows the number of probes specific to *S*. *flexneri*, *A*. *fumigatus* and vaccinia virus detected out of the total number of probes designed to each respective target. The array uses a threshold of signal intensities greater than the 99^th^ percentile of the negative controls and at least 20% of probes detected to determine a positive detection. *S*. *flexneri* was positively detected at 100 genome copies and above, but not at 10 genome copies. The number of probes detected at 100 copies was 2,185 out of 7,398 probes expected, or about 29%. The number of probes detected at 1,000 copies was 3,781 out of 8,009 probes expected, or about 47%. Please note that the number of probes expected between the 100 vs 1,000 genome copy samples have a small difference. This is due to two different target strains (both draft sequences but very close) which were detected. Similarly, *A*. *fumigatus* was also detected at 100 or more genome copies. The number of probes detected at 100 copies was 55 out of 168 probes expected, or about 33%. The number of probes detected at 1,000 copies was 81 out of 168 probes expected, or about 48%. Vaccinia virus was only detectable at 1,000 or more copies. The number of probes detected at 1,000 copies was 78 out of 293 probes expected, or about 26%. The number of probes detected at 10,000 copies was 148 out of 286 probes expected, or about 52%. The lower detection sensitivity is possibly due to the small number of probes designed for this virus. However, while the total number of probes designed for *A*. *fumigatus* was smaller than that for vaccinia virus, *A*. *fumigatus* was detected at 100 copies.

**Table 3:** Analysis of the sensitivity of bacterial, fungal and viral target detection on the Axiom Microbiome Array. The genomic DNA was spiked into soil DNA background. The average number of probes detected was an average of two replicates with standard deviation.

Target spiked	Genome copy number	Target detected as	Average # of probes detected /expected^a^
*Aspergillus fumigatus*	10	Not detected	NA
	100	*A*. *fumigatus* Af10	55±1 /168
	1000	*A*. *fumigatus* Af10	81±0 /168
	10000	*A*. *fumigatus* Af10	83±0 /168
*Shigella flexneri*	10	Not detected	NA
	100	*S*. *flexneri* 2930–71 draft (50 frags)	2185±42 /7398
	1000	*S*. *flexneri* II:(3)4 7(8) draft (196 frags)	3781±125 /8009
	10000	*S*. *flexneri* II:(3)4 7(8) draft (196 frags)	4027±88 /8009
Vaccinia lister	100	Not detected	NA
	1000	Vaccinia virus strain DUKE	78±4 /293
	10000	Vaccinia virus	148±1 /286

^a^ The number of probes detected vs expected at different concentrations could be different if a different target strain was predicted by the array.

In addition to the spiked organism, other organisms were detected by the array, possibly from the soil background. In *S*. *flexneri* spiked samples, an additional 29 bacterial species from a total of 15 families (including one unknown bacterial family) were detected (S1 Table). *S*. *flexneri* had the highest conditional scores among all species detected at 100 copies and higher concentrations based on the MiDAS analysis. In *A*. *fumigatus* spiked samples, an additional 27 bacterial species from a total of 18 families (including one unknown bacterial family) were detected (S1 Table). In vaccinia virus strain Lister spiked samples, an additional 24 bacterial species from a total of 16 families (including one unknown bacterial family) were detected (S1 Table). Vaccinia Lister had the highest conditional scores among all species at the 10,000 copies concentration. For the samples where the spiked species did not have the highest conditional score, *Bradyrhizobium* sp. had the highest score instead. *Bradyrhizobium* is a gram-negative soil bacterium, which is expected given soil DNA was used as the background for the spiked tests.

### Analysis of Metagenomic Control Material on the Axiom Microbiome Array

The detection sensitivity of the Axiom Microbiome Array was evaluated using the ATCC Metagenomic Control Material for Pathogen Detection. The genome sizes of the mix range from 1.85Mbp in *Streptococcus pyogenes* to 6.30 Mbp in *Pseudomonas aeruginosa*. Table 4 lists the pathogens in descending order of their percent content in the mix, from 28.9% in *Streptococcus pneumoniae* to 0.1% in *Acinetobacter baumannii*. The array detected 11 out of the 11 pathogens from this mix at the species level, covering a dynamic range from 100,282 copies to 389 copies. The array also matched 10 out of the 11 target sequence. The only pathogen that did not match was *Streptococcus pneumoniae* strain Spain 23F-1 (ATCC 700669); instead *Streptococcus pneumoniae* GA41565 draft (9 frags) was detected. In MiDAS, draft genomes can have higher conditional scores than curated genomes when multiple chromosomes and plasmids are separated into different target sequences (Thermo Fisher Axiom Microbiome User Manual). Detailed array data are provided in S1 Table.

**Table 4:** Axiom Microbiome Array testing results of ATCC Metagenomic Control Material (ATCC MSA-4000).

Organisms in the sample mix	Percentage	Copies/ng^a^	Correct species detected?	Axiom Microbiome Array target detection (chromosome and plasmid)
*Streptococcus pneumoniae* strain Spain 23F-1 (ATCC 700669)	28.90	120,607	Yes	*Streptococcus pneumoniae* GA41565 draft (9 frags)
*Neisseria meningitidis* strain FAM18 (ATCC 700532)	28.90	117,950	Yes	*Neisseria meningitidis* FAM18 chromosome
*Staphylococcus aureus* subsp. *aureus* (MSSA) strain TCH959 (ATCC BAA-1718)	14.40	47,647	Yes	*Staphylococcus aureus* subsp. *aureus* USA300_TCH959 plasmid pUSA300HOUMS
*Streptococcus pyogenes* strain SF370 (ATCC 700294)	7.20	36,057	Yes	*Streptococcus pyogenes* SF370 chromosome
*Klebsiella pneumoniae* subsp. *pneumoniae* strain MGH78578 (ATCC 700721)	14.40	25,172	Yes	*Klebsiella pneumoniae* KP4-R draft (111 frags), *Klebsiella pneumoniae* subsp. *pneumoniae* MGH 78578 plasmid pKPN5
*Streptococcus agalacitae* strain 2603 V/R (ATCC BAA-611)	2.90	12,157	Yes	*Streptococcus agalactiae* 2603V/R chromosome
*Escherichia coli* strain CFT073(ATCC 700928)	1.40	2,760	Yes	*Escherichia coli* CFT073 chromosome
*Staphylococcus aureus* subsp. *aureus* (MRSA) strain FPR3757 (ATCC BAA-1556)	0.70	2,316	Yes	*Staphylococcus aureus* subsp. *aureus* USA300_FPR3757 plasmid pUSA03
*Enterococcus faecalis* strain V583 (ATCC 700802)	0.70	2,140	Yes	*Enterococcus faecalis* V583 draft (10 frags)
*Pseudomonas aeruginosa* strain PAO1-LAC (ATCC 47085)	0.30	441	Yes	*Pseudomonas aeruginosa* PAO1 chromosome
*Acinetobacter baumannii* (ATCC 17978)	0.10	233	Yes	*Acinetobacter baumannii* ATCC 17978 chromosome, pAB2

^a^Copies per ng was calculated based on the molecular weight of each organism.

**Fig 1:**
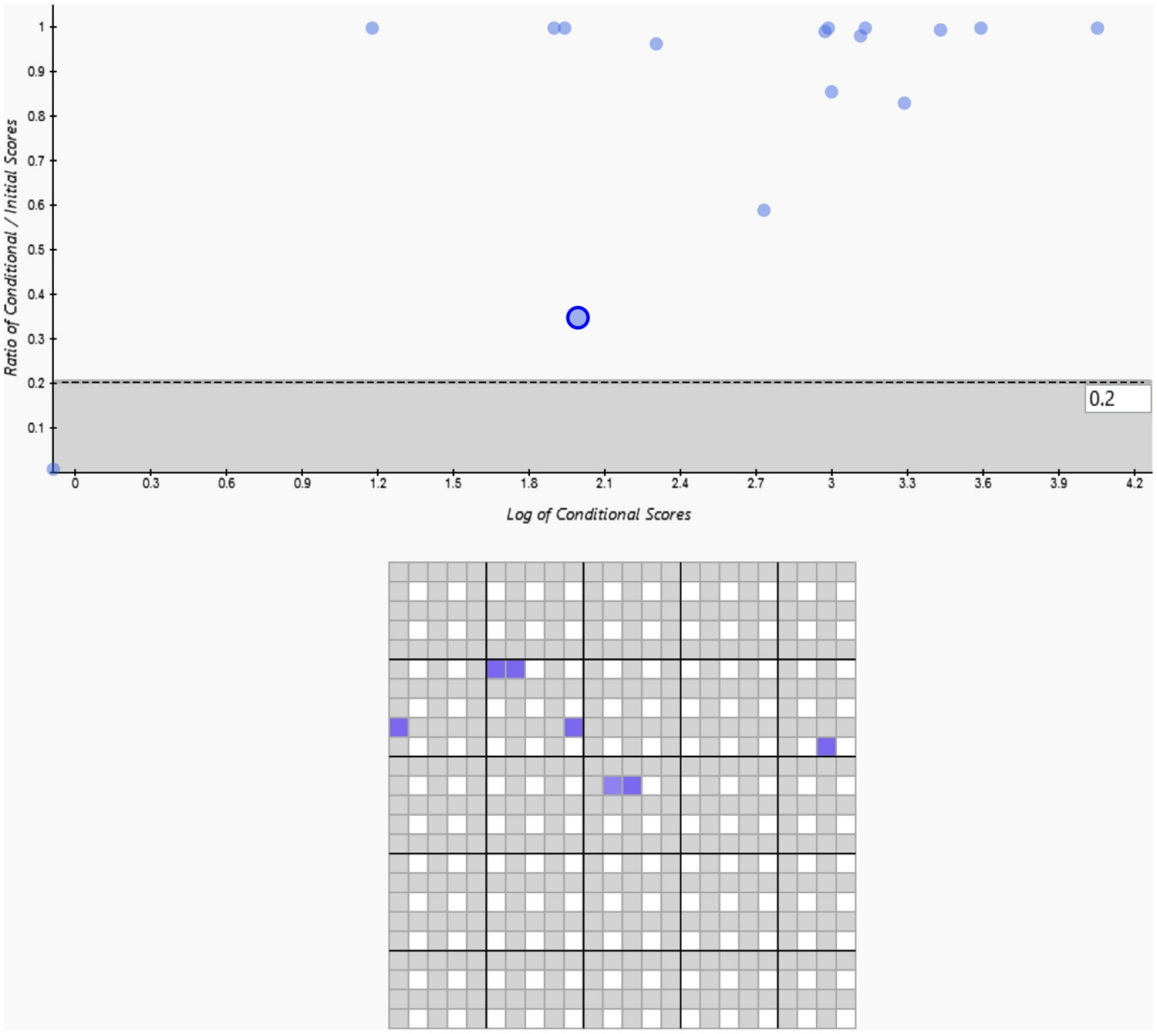
The log of conditional scores vs. ratio of conditional/initial scores graph of the ATCC Metagenomic Control Material. The x-axis (Log of Conditional Scores) provides information on the relative contribution of each target to the model provided by Axiom MiDAS, while the y-axis provides the ratio of the actual probe-level data used to contribute to the model relative to its possible maximal contribution. The default threshold is 0.2. Each circle represents one unique target sequence. The circle highlighted represented *Klebsiella pneumoniae* subsp. pneumoniae MGH 78578 plasmid pKPN. All other genomic targets were detected at a ratio of >0.6.

The log of conditional scores vs. ratio of conditional/initial scores graph of the ATCC Metagenomic Control Material is shown in Fig 1. The x-axis (Log of Conditional Scores) provides information on the relative contribution of each target to the model provided by Axiom MiDAS, while the y-axis provides the ratio of the actual probe-level data used to contribute to the model relative to its possible maximal contribution. The default threshold is 0.2. This setting suggests that targets having 80% or more of their initial log likelihood score explained by prior hits on the array are unlikely to be present as distinct targets in the sample. Each circle represents one unique target sequence. The highlighted circle represents *Klebsiella pneumoniae* subsp. pneumoniae MGH 78578 plasmid pKPN. All other genomic targets were detected at a ratio of greater than 0.6.

**Fig 2:**
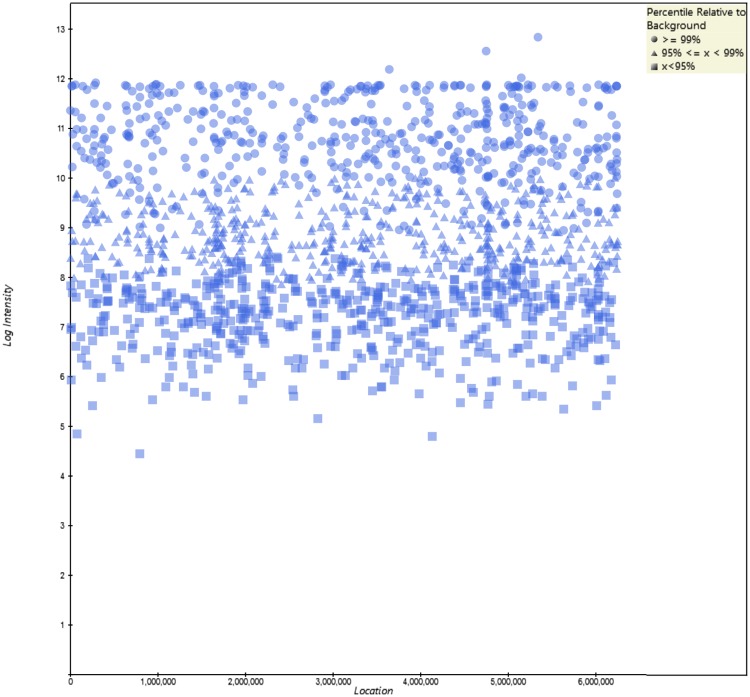
The *Pseudomonas aeruginosa* probes detected from the ATCC Metagenomic Control Material across its genome. This graph is plotted as the log intensity of probes detected for a given target versus the position of the probe on the target sequence. Only probes that are above 99% of background probes (in round dots) are considered positive. The probes that are between 95% and 99% (in triangle) and below 95% (square) are not considered positive.

The probe location plots from MiDAS are shown in Fig 2 using *Pseudomonas aeruginosa* probes detected from the ATCC Metagenomic Control Material as an example. This graph plots the log intensity of probes detected for a given target vs the positions of the probes on the target sequence. In our analysis, only probes that are above 99% of background probes (in round dots) are considered positive. The probes that are between 95% and 99% (in triangle) and below 95% (square) are not considered positive.

Next, we tested the ATCC 20 Strain Staggered Mix Genomic Material. The genome sizes of these bacteria range from 1.67 Mbp in *Helicobacter pylori* to 6.30 Mbp in *Pseudomonas aeruginosa*. The results are summarized in Table 5. This table lists the pathogens from the mix in descending order of the percent content in the mix, from 18% in *Streptococcus mutans* to 0.02% as in *Bacteroides vulgatus*. The genomic DNA copy numbers range from 82,149 to 36 copies per ng. The array correctly detected 17 out of 20 bacteria at the species-level. Sixteen of the 17 bacterial DNA have copy numbers above 400 copies/ng. One of the 17 bacterial DNA detected by the array, *Bacteroides vulgatus*, has genomic DNA at 36 copies, which shows that the array is capable at detecting lower concentrations of selected species. *Deinococcus radiodurans* was positively detected at 70 genome copies, though only plasmids CP1 and MP1 and not its chromosome sequence was detected. The three DNAs that were not detected were *Bifidobacterium adolescentis* (ATCC 15703), *Actinomyces odontolyticus* (ATCC 17982), and *Enterococcus faecalis* (ATCC 47077). All three of these DNAs are at the lowest percentage (0.02%) of the overall mix, and between 61–89 genome copies. This result shows that the array can cover a dynamic range of three orders of magnitude. Detailed array data are provided in S1 Table.

**Table 5:** Axiom Microbiome Array results of ATCC 20 Strain Staggered Mix Genomic Material (ATCC MSA-1003).

Organisms in the sample mix	Percentage	Copies/ng	Correct species Detected?	Axiom Microbiome Array target detection (chromosome and plasmid)
*Streptococcus mutans* strain UA159 (ATCC 700610)	18.00	82,149	Yes	*Streptococcus mutans* UA159 chromosome
*Porphyromonas gingivalis* (ATCC 33277)	18.00	71,266	Yes	*Porphyromonas gingivalis* ATCC 33277
*Staphylococcus epidermidis* (ATCC 12228)	18.00	69,485	Yes	*Staphylococcus epidermidis* ATCC 12228 chromosome, plasmid pSE-12228-03, -04, -05, -06
*Rhodobacter sphaeroides* (ATCC 17029)	18.00	40,378	Yes	*Rhodobacter sphaeroides* ATCC 17029 chromosome 2, pRSPH01, *Rhodobacter sphaeroides* WS8N draft (4 frags)
*Escherichia coli* Strain MG1655 (ATCC 700926)	18.00	35,482	Yes	*Escherichia coli* str. K12 substr. MG1655
*Streptococcus agalactiae* strain 2603 V/R (ATCC BAA-611)	1.80	7,546	Yes	*Streptococcus agalactiae* 2603V/R chromosome
*Staphylococcus aureus* strain FPR3757 (ATCC BAA-1556)	1.80	5,956	Yes	*Staphylococcus aureus* W89268 draft (24 frags)^a^
*Bacillus cereus* (ATCC 10987)	1.80	3,082	Yes	*Bacillus cereus* ATCC 10987, plasmid pBc10987
*Clostridium beijerinckii* strain SA-1 (ATCC 35702)	1.80	2,779	Yes^b^	*Clostridium beijerinckii* NCIMB 8052 chromosome^c^
*Pseudomonas aeruginosa* strain R. Hugh 813 (ATCC 9027)	1.80	2,647	Yes	*Pseudomonas aeruginosa* PA7 chromosome
*Helicobacter pylori* strain 26695 (ATCC 700392)	0.18	999	Yes	*Helicobacter pylori* 26695–1 DNA
*Lactobacillus gasseri* (ATCC 33323)	0.18	882	Yes	*Lactobacillus gasseri* ATCC 33323 chromosome
*Neisseria meningitidis* strain MC58 (ATCC BAA-335)	0.18	735	Yes	*Neisseria meningitidis* 9757 draft (32 frags)
*Propionibacterium acnes* (ATCC 11828)	0.18	651	Yes	*Propionibacterium acnes* ATCC 11828 chromosome^d^
*Acinetobacter baumannii* (ATCC 17978)	0.18	419	Yes	*Acinetobacter baumannii* ATCC 17978 chromosome, plasmid pAB2
*Bifidobacterium adolescentis* (ATCC 15703)	0.02	89	No	NA
*Actinomyces odontolyticus* (ATCC 17982)	0.02	79	No	NA
*Deinococcus radiodurans* (ATCC BAA-816)	0.02	70	Yes	*Deinococcus radiodurans* R1 plasmid CP1, MP1
*Enterococcus faecalis* (ATCC 47077)	0.02	61	No	NA
*Bacteroides vulgatus* (ATCC 8482)	0.02	36	Yes	*Bacteroides vulgatus* ATCC 8482 chromosome

^a^ In 1 out of 2 replicates, this was detected as *Staphylococcus aureus* M79686 draft (25 frags)

^b^ Detected in 1 out of 2 replicates.

^c^ *Clostridium beijerinckii* SA-1 was derived by directed evolution from *C*. *beijerinckii* NCIMB8052.

^d^ In 1 out of 2 replicates, this was detected as *Propionibacterium acnes* J139 draft (7 frags).

Out of the 17 species that were correctly identified by the array, 14 of these bacteria were correctly identified at the target sequence level. For the remaining species, the correct target sequence was identified by the MiDAS software as a “secondary hit”, i.e. an alternative but lower-scoring explanation for part of the observed probe data. For example, the correct *Staphylococcus aureus* strain FPR3757 was detected as a secondary hit with a conditional log score of 4644.7, very close to the conditional score 4758.0 reported for the “primary hit” *S*. *aureus* W89268 strain draft genome (S1 Table). *Neisseria meningitidis* strain MC58 was also detected as a secondary hit, with a log conditional score of 1132.94, very close to the *Neisseria meningitidis* 9757 draft with a log conditional score of 1134.43. As explained earlier, draft genomes can have higher conditional scores than curated genomes where multiple chromosomes and plasmids are separated into different target sequences. A secondary hit often shares almost all the same probes as the primary hit. *Pseudomonas aeruginosa* strain R. Hugh 813 (ATCC 9027) was identified as the *Pseudomonas aeruginosa* PA7 strain.

### Detection of viruses and bacteria from pig clinical samples

Pig clinical samples were analyzed on the Axiom Microbiome Array. The detailed microarray results of all the clinical samples tested are summarized in S2 Table (serum and tonsil samples) and S3 Table (fecal samples). The array data from one representative fecal sample is shown in Table 6. Log of conditional scores, numbers of probes expected, and number of probes detected are shown in the table with the corresponding family and species detected. A total of 13 microbial families were detected in this sample, representing 33 unique species. Bacterial families that are often observed in the gut microbiome such as *Lactobacillaceae*, *Prevotellaceae*, *Veillonellaceae*, *Streptococcaeae*, and *Ruminococcaceae* were found. A viral family, Parvoviridae was also found.

**Table 6:** Microbial species detected by Axiom Microbiome Array from a pig fecal sample (No. 289). The pig was experimentally infected with PRRSV and PCV-2. Species are listed in decreasing order of conditional score.

Conditional score	Num. probes expected	Num. probes detected	Family	Species
569.52	417	203	*Lactobacillaceae*	*Lactobacillus reuteri*
467.97	281	161	*Lactobacillaceae*	*Lactobacillus acidophilus*
322.29	130	91	*Parvoviridae*	*Ungulate bocaparvovirus 5*
236.03	114	71	*Prevotellaceae*	*Prevotella copri*
231.67	273	106	*Lachnospiraceae*	*Dorea longicatena*
138.95	243	84	*Streptococcaceae*	*Streptococcus equinus*
126.67	76	57	*Parvoviridae*	*Porcine bocavirus 5*
122.14	210	124	*Lactobacillaceae*	*Lactobacillus johnsonii*
118.74	146	72	*Prevotellaceae*	*Prevotella stercorea*
116.69	223	80	*Ruminococcaceae*	*Faecalibacterium prausnitzii*
114.83	59	35	*Lactobacillaceae*	*Lactobacillus acidophilus*
110.74	94	55	*Lactobacillaceae*	*Lactobacillus fermentum*
106.99	91	42	*Veillonellaceae*	*Megasphaera elsdenii*
83.87	106	46	*Acidaminococcaceae*	*Phascolarctobacterium succinatutens*
77.01	89	36	Parvoviridae	Ungulate bocaparvovirus 2
70.58	106	38	*Erysipelotrichaceae*	*Catenibacterium mitsuokai*
66.15	111	39	*Veillonellaceae*	*Mitsuokella multacida*
62.57	42	23	*Lactobacillaceae*	*Lactobacillus sakei*
59.58	96	63	*Parvoviridae*	*Porcine bocavirus*
48.1	50	21	*Bacteria_unknownfamily*	*uncultured bacterium*
40.86	113	49	*Prevotellaceae*	*Prevotella sp*. *RM4*
37.02	67	31	*Lactobacillaceae*	*Lactobacillus amylovorus*
34.71	319	110	*Lachnospiraceae*	*Roseburia intestinalis*
31.3	69	42	*Parvoviridae*	*Ungulate bocaparvovirus 5*
29.94	94	35	*Ruminococcaceae*	*Ruminococcus callidus*
29.94	19	10	*Streptococcaceae*	*Streptococcus suis*
29	94	32	*Eubacteriaceae*	*[Eubacterium] eligens*
25.7	73	27	*Veillonellaceae*	*Dialister succinatiphilus*
21.26	106	72	*Parvoviridae*	*Ungulate bocaparvovirus 5*
19.41	75	22	*Oscillospiraceae*	*Oscillibacter sp*. *1–3*
18.63	152	51	*Eubacteriaceae*	*Eubacterium rectale*
9.13	105	75	*Parvoviridae*	*Porcine bocavirus*
6.06	91	35	*Prevotellaceae*	*Prevotella paludivivens*
3.67	55	14	*Streptococcaceae*	*Streptococcus agalactiae*
3.54	19	9	*Aerococcaceae*	*Abiotrophia defectiva*
2.69	151	74	*Lactobacillaceae*	*Lactobacillus vaginalis*
2.39	69	35	*Lactobacillaceae*	*Lactobacillus amylovorus*
0.27	30	14	*Aerococcaceae*	*Abiotrophia defectiva*

We evaluated the reproducibility of results from replicate samples tested on the array. Table 7 summarizes the species level consensus calls from three replicates of each pig fecal sample. Overall, a 95% consensus hit rate and 93% consensus precision rate were obtained. These samples passed the aggregate reproducibility specifications on species from MiDAS analysis. Consensus of target sequence level calls were also analyzed (data not shown) and the specs also passed.

**Table 7:** Consensus of species level detection on the Axiom Microbiome Array from three technical replicates of 8 pig fecal samples.

Samples	Consensus Detected	Consensus Not Detected	Not in consensus	CHR (Consensus Hit Rate)	CP (Consensus Precision)	CHR Average	CP Average
289–1	28	0	0	1.00	1.00	0.94	0.98
289–2	24	4	1	0.86	0.96		
289–3	27	1	1	0.96	0.96		
290–1	33	2	2	0.94	0.94	0.93	0.92
290–2	33	2	5	0.94	0.87		
290–3	32	3	2	0.91	0.94		
292–1	38	1	7	0.97	0.84	0.96	0.91
292–2	37	2	1	0.95	0.97		
292–3	37	2	3	0.95	0.93		
294–1	39	3	2	0.93	0.95	0.91	0.92
294–2	37	5	5	0.88	0.88		
294–3	39	3	3	0.93	0.93		
321–1	36	1	9	0.97	0.80	0.91	0.87
321–2	33	4	4	0.89	0.89		
321–3	32	5	2	0.86	0.94		
350–1	27	5	5	0.84	0.84	0.93	0.88
350–2	31	1	4	0.97	0.89		
350–3	31	1	3	0.97	0.91		
351–1	27	4	2	0.87	0.93	0.94	0.94
351–2	31	0	1	1.00	0.97		
351–3	29	2	3	0.94	0.91		
354–1	29	5	5	0.85	0.85	0.93	0.89
354–2	34	0	2	1.00	0.94		
354–3	32	2	5	0.94	0.86		

Next, we analyzed three serum samples and three tonsil samples on the Axiom Microbiome Array. These samples were from a previous study of experimental infection of PRRSV and PCV-2 viruses and they were evaluated on the Agilent version of the LLMDA (v.7) [18]. The detection of PRRSV, PCV-2 and other viruses in these samples are summarized in Table 8. The results between the LLMDA v.7 and the Axiom Microbiome Array are compared in terms of the viruses detected from these samples. For sample analysis on the Axiom Microbiome Array, DNA and RNA samples were extracted separately and tested on the array separately. PCV-2 was detected from both DNA and RNA extractions. Since PRRSV is an RNA virus, it was only detected from the RNA. The results listed on Table 8 are from RNA extracts of these samples. In serum samples 145, 147 and 148, both PCV-2 and PRRSV were detected by both the LLMDA Agilent array and the Axiom Microbiome Array. The PCV-2 genome copies were between 5.2 to 6.8 logs, while the PRRSV genome copies were between 2.8 to 5.6 logs. For tonsil samples, the LLMDA v.7 detected PCV-2 from 170 and 173, but not from 171. However, PCV-2 from sample 171 was detected on the Axiom Microbiome Array. The serum samples from 170, 171 and 173 had PCV-2 genome copies at 6.1, 1.6 and 2.4 logs. It is likely that the tonsil samples from the same pigs had a similar PCV-2 concentration, but PCR was not performed on the tonsil samples. The LLMDA v.7 did not detect PRRSV in samples 170, 171 and 173. Using the Axiom array, PRRSV was detected in sample 173, but not in 170 and 171. The PRRSV genome copies were very low (< 1 log) in serum samples from the same pigs. In addition to PCV-2 and PRRSV, a few other viruses were detected from both serum and tonsil sample types.

**Table 8:** Viruses detected from pig serum and tonsil samples on the Axiom Microbiome Array and comparison to the Agilent version of the LLMDA [18]. The results for the Agilent array were generated using total nucleic acid. The results from Axiom Microbiome Array were generated using RNA from these samples.

Sample ID	Sample type	Agilent LLMDA	Axiom Microbiome Array
**145**	Serum	A, B, C, F	A, B, C, F
**147**	Serum	A, B, D, F, G	A, B, D, F, H
**148**	Serum	A, B, D, F	A, B, D, F
**170**	Tonsil	A, F	A, F
**171**	Tonsil	F	A, F
**173**	Tonsil	A, F	A, B, F

A: Porcine circovirus 2 (PCV-2); B: Porcine reproductive and respiratory virus (PRRSV); C: Torque teno sus virus 1a (TTSuV-1a); D: TTSuV-1b; E: TTSuV-k2; F: Porcine type-c oncovirus retrovirus A and/or C; G: Porcine bocavirus 4–1; H: porcine bocavirus 3 isolate.

## Discussion

Microarrays, along with PCR and DNA sequencing are effective methods for microbial detection and discovery using nucleic acid samples. While microarrays are not as sensitive as PCR assays [18, 20], a single array can analyze more than 12,000 species of microbes as compared to at most a few tens of regions in the largest multiplexed PCR assays.

The Axiom Microbiome Array is the first high-throughput microarray platform for comprehensive analysis of all sequenced microbial species in 24- or 96-well format. This platform is more cost-effective to screen large number of samples as compared to metagenomic sequencing. However, the Axiom Microarray Array was designed for taxonomy identification. The array does not provide any functional characterization of genes and other sequence variations. For detection of genes, mutations and unknown organisms, metagenomic sequencing is more suitable than microarray. Additionally, the Axiom Microbiome Array was designed based on all sequenced microbial genomes as of October 2014. Newly sequenced microbial genomes that were not available at the time of array design are not represented on the array.

When compared to 16S rRNA gene sequencing, the Axiom Microbiome Array have probes to detect viruses, fungi, protozoa and archaea in addition to bacteria. The Axiom Microbiome Array was designed against both conserved regions and unique regions of the microbial genomes, therefore should provide higher specificity than 16S rRNA sequencing. However, for analysis of unknown bacteria, 16S rRNA sequencing is more suitable than the Axiom Microbiome Array since 16S rRNA gene is conserved.

The detection sensitivity of the Axiom Microbiome Array was evaluated by using serial dilutions of a DNA virus, vaccinia virus Lister, a bacterium, *S*. *flexneri*, and a fungus, *A*. *fumigatus*. The Axiom Microbiome Array detected *S*. *flexneri* and *A*. *fumigatus* at 100 genome copies/reaction. This sensitivity is close or identical to the lower-plex Agilent format of the LLMDA [18]. For vaccinia virus Lister DNA, the array detected 1,000 genome copies. This detection sensitivity is lower than for the lower-plex LLMDA array previously reported, which was 100 copies when random whole genome amplification was used [11].

The Axiom Microbiome Array can detect a large dynamic range, from ~30 to ~120,000 copies of a mixture of bacterial DNAs. Another important feature of the Axiom Microbiome Array is its ability to make species level calls for the sequenced bacteria, viruses, fungi, archaea and protozoa that are represented on the array. The Axiom Microbiome Array accurately detected 11/11 bacteria from the Metagenomic Control Material at the species level. The species level specificity of array provides valuable information for diagnosis and surveillance of pathogens in clinical and environmental samples. The strain-level detection by the Axiom Microbiome Array was not evaluated in this study, however, since the array was designed to detect specific genomic regions of the sequences represented on the array, strain-level detection should be feasible. Future studies should include testing of closely related near neighbor species and a number of strains from the same species.

The lower cost and higher-throughput capabilities of the array make it a suitable technology for routine surveillance and diagnosis of large numbers of veterinary samples, especially for screening of unknown pathogens and polymicrobial infections. In this study, swine fecal, serum and tonsil samples from pigs experimentally infected with PCV-2 and PRRSV were tested on the Axiom Microbiome Array. A range of bacterial families including some that are generally observed in fecal microbiome such as *Bacteroidaceae*, *Bifidobacteriaceae*, and *Enterobacteriaceae* were detected from pig fecal samples. The results of microbiome analysis were reproducible across triplicate samples. The bacterial families confirmed the findings from previous studies using the Agilent version of the LLMDA v.7 to determine microbiome content from pigs with different clinical outcomes [17, 19]. For viral detection, the Axiom Microbiome Array detected Torque teno suis virus (TTSuV) and porcine oncoviruses, both of which are commonly found in pigs and were also identified in the clinical samples [27, 28]. When compared to the results of the LLMDA v.7 [18], the Axiom Microbiome Array achieved similar or identical results in terms of the number of species that were positively detected.

The Axiom Microbiome Array and the Agilent arrays have different probe design strategies and the detection chemistry. The Agilent array uses 60-mer oligos as probes, while the Axiom array uses 35-mer oligos. The longer probe may provide better hybridization with target DNA, however the Axiom array contains much higher probe density than the Agilent platform. Both array platforms employ fluorescence-based detection. Agilent arrays use the Cy-3 labeled random primers to label target DNA before hybridization on the array. The Axiom platform performs hybridization of target DNA to the array first, then multi-colored labels are ligated to the probes and subsequently stained for fluorescence detection. The different detection chemistry between the two array platforms could result in different detection sensitivities of various targets.

DNA extraction methods also affect the detection sensitivity and accuracy, especially from complex samples. This study utilized the Trizol LS reagent and PowerViral environmental DNA/RNA isolation kit for the extraction of pig clinical samples [17, 18]. In a recent cow and sheep microbial communities study, 15 DNA extraction methods were compared and none of the DNA extraction methods resulted in 100% comparable community compositions [29]. Another study compared the DNA extraction methods for analysis of human oral bacterial and fungal communities. It was found that some extraction methods were better suited for fungal, and vice versa [30]. Therefore, when choosing a DNA extraction method, factors such as yield, quality for downstream analysis and variability between different researchers and different machines should be considered.

As with any technology based on nucleic acid detection, the capabilities of microarrays are limited by the genome sequence information available at the time of design. It is estimated that we have only sequenced 1 x 10^−22^% of the total DNA on Earth, and ~1,400 or less than 1% of the total number of microbial species on the planet are human pathogens [31]. As more and more genomes are sequenced, the microarrays should be updated to include the additional sequences to better detect and identify microbial species from clinical and environmental samples.

## Conclusion

The Axiom Microbiome Array provides a cheaper and higher-throughput approach for microbiome analysis and pathogen detection. The results from this study have demonstrated that the array can accurately detect members of the mock microbial communities with three orders of magnitude dynamic range, achieving 100% accuracy in species identification. The Axiom Microbiome Array presents a new opportunity for screening of thousands of microbial species at a fraction of the cost of metagenomic sequencing. The array has the potential for rapid screening of bacterial and viral infections from human and veterinary samples, and surveillance of pathogens from environmental samples. Future designs of the array could include probes to detect functional and metabolic genes, which will enhance the application of the microarray beyond taxonomy identification. Further advances in array technology such as faster hybridization times, more integrated sample preparation, amplification, hybridization and washing system, and label-free direct-detection methods will broaden the applications of microarrays even further. Ultimately, microarrays could potentially be advanced into point-of-care devices which deliver results in a few hours.

## Supporting information

S1 Table Axiom Microbiome Array results of the two ATCC microbiome standards, *S*. *flexneri*, *A*. *fumigatus* and Vaccinia lister DNA spiked samples and a negative control sample (no-template-control). (XLSX)

S2 Table Axiom Microbiome Array results of pig serum and tonsil samples (145, 147, 148, 170, 171, 173) provided by Kansas State University. The results from each sample are separated by tabs.
(XLSX)

S3 Table Axiom Microbiome Array results of pig fecal samples (289, 290, 292, 294, 321, 350, 351, 354 in duplicates) provided by Kansas State University. (XLSX)
